# Drainage of the Retention Catheter in Operations on the Genito-Urinary Tract

**Published:** 1907-09

**Authors:** L. Sage Hardin


### DRAINAGE BY THE RETENTION CATHETER IN OPERATIONS ON THE GENITO-URINARY TRACT.

By Dr. L. Sage Hardin.

Until 1902, I had had no experience with the retention catheter, but to the contrary, had dressed perineal drains after urethrotomies and suprapubic wounds, after cystotomies for weeks and sometimes months and in one case a perineal fistula persisted for one half year, all this time the patients were being subjected to continuous soiling of the bedding and their linen, and the urethrotomies were followed by chills when I began passing sounds at as small a number as 22 or 23F, although the urethra had been cut to a No. 32.

In 1902 the picture changed, when a man 72 years old came under Dr. McRae's care for a prostatic enlargement, after futile attempts had been made for three days to catheterize him, to my gratification a 22F. passed into his bladder at the first trial, and for fear I would be unable to get another in, this one remained for five days. Permanent drainage continued in this case until operation thirty-four days, then afterwards twenty-three days, fifty-seven days in all.

Since then, we have used this method in fourteen protatectomies, twenty urethrotomies, three supra pubic cystotomies, for stone; five case of cystitis; two hysterectomies; one case of hypospadias; one gun-shot wound of urethra, forty-six cases in all.

The catheter instead of causing distress often gives entire relief, and when pain is caused, it is due to improper application of the method. During the first few hours, there is some inconvience, but this quickly subsides, and the catheter may then be continued for weeks.

The success of this method depends greatly upon the proper placing of the catheter and careful attention.

The catheter should be soft rubber, but rather heavy and introduced with the eye forward, so as not to become occluded by the base of the bladder; this can be assured by marking the base of the tube in the line of the eye; when introduced, the catheter should be well beyond the vesical sphincter, otherwise it may be forced out by a spasm of the same, causing pain and retention. For renewing a catheter, a guide, to keep it against the root of the penis is of a great service. A solution of boric acid or saline should be introduced into the bladder and while it is returning, adjustments may be made as to the depth of the tube and where it should be maintained.

In men, I have a small safety pin passed through the catheter on each side, just in front of the meatus, and attached to narrow strips of adhesive plaster, which pass along each side of the penis, to the groin, and fastened by adhesive around the dor-retaining the catheter.

The tube can then be adjusted or removed without changing the dressing. In the female, Pezzer's catheter is easily introduced by passing an instrument into the eye and making tension on the catheter.

In prostatic cases, where the eyes becomes occluded by the loose tissue, or is forced into the urethra, it is best retained for the first few days, by a linen or silk thread passed through the tip of the catheter, inside of the bladder, and brought through the supra pubic wound, and there fastened by a safety pin. The supra-pubic drain we retain by taking a section of tubing sufficiently long to reach from the skin to bladder and passing through this a Pezzer's retention catheter. A pin is placed through tube above the skin to prevent tube from slipping inward. This method gives drainage and irrigation above and below.

In case of prostatectomies or urethrotomies, the case should be seen several times within the first twelve hours and any stoppage of the flow corrected by adjusting the catheter, or dislodging a blood clot in the eye, by passing in an irrigating solution; the latter may frequently be prevented by injecting adrenalin into the urethra before the catheter is introduced.

Following this, in any case, there shonld be a daily irrigation of the bladder through the tube and of the urethra around it.

I have never seen any advantage in frequent changing of the catheter, however, where drainage is continued for some time, it should be changed once in five or seven days for cleanliness. There is a mechanical urethritis established which quickly subsides after the catheter is removed. Only in one case have I seen an erection while catheter was in, and not a case of orchitis or epididymitis. Drainage by the retention is of service in urethrotomies; by allowing a small perineal wound which may be sutured, a small drain being placed in the posterior angle of the wound for twenty-four hours, primary union usually occurs in a week; by controlling urethral bleeding; maintaining the catheter, and softening the urethra, so that after removal of the catheter a 28, 30 or 32 F. sound may be passed easily without the fear of a chill.

In prostatectomies and cystotomies by permitting free irrigation and allowing the abdominal wound to heal rapidly without undue soiling of the patient.

In vaginal hysterectomies or operations upon the vulva where it is necessary to protect our dressings.

In plastic operations upon the penis, cystitis, retention of urine, or excessively frequent urination, and in injuries to the urethral canal, etc.

